# They called it the ‘abominable crime’: an analysis of heterosexual support for anti-gay laws in Barbados, Guyana and Trinidad and Tobago

**DOI:** 10.1007/s13178-015-0209-6

**Published:** 2015-10-15

**Authors:** Mahalia Jackman

**Affiliations:** The Cathie Marsh Institute for Social Research, School of Social Sciences, University of Manchester, 2.11 Humanities Bridgeford St Building, Manchester, M13 9PL UK

**Keywords:** Anti-gay laws, Caribbean, Gay rights, Attitudes towards homosexuals, Buggery laws, Homonegativity, Homophobia

## Abstract

The aim of this study was to evaluate support for current buggery/sodomy laws in three Caribbean countries—Barbados, Guyana and Trinidad and Tobago. To complete this task, data from the 2013 Caribbean Development Research Services (CADRES) ‘Attitudes towards homosexuals’ surveys were employed. The data analysis revealed that a majority of heterosexuals in the sample generally supported the maintenance and enforcement of the anti-gay laws, and the main predictors of said support were race, country of residence, religiosity, interpersonal contact and beliefs about the origins of homosexuality.

## Introduction

Over the last few decades, there has been an unprecedented rise in the acceptance of homosexuals. Specifically, a number of countries have decriminalised same-sex sexual acts and now extend legal rights towards homosexuals such as the right to adopt, marry or engage in civil unions. Most notable was the recent decision by the United States (US) Supreme Court to make same-sex marriage legal throughout the US. The extension of marital rights to homosexuals in the US is considered a remarkable feat in the gay rights movement, as the US currently stands as one of the most influential countries in the world. However, the trend towards socio-legal acceptance of homosexuality is not a global phenomenon: homosexuality remains ‘taboo’ in many countries. According to the 2015 International Lesbian, Gay, Bisexual, Trans and Intersex Association’s State Sponsored Homophobia report, over 70 countries still penalise private consensual same-sex intimate acts (Carroll and Itaborahy 2015), among which are Barbados, Guyana and Trinidad and Tobago (henceforth T&T).

In the last decade, the continued criminalisation of private same-sex sexual acts has received considerable international attention, and like many other countries, Barbados, Guyana and T&T have come under fire for their anti-gay laws. The choice to keep these laws on the books and put longstanding international relations at risk suggests that strong domestic social forces could be at play. Specifically, under a theory of legal moralism, if a common morality is held by the majority, policymakers may opt to put/keep legislations in place to uphold said morality and remain in public favour (Beauchamp 1986; Burstein 1998, 2003). Thus, a perceived lack of public appetite for homosexual law reform may encourage policymakers to keep the laws on the books. Against this backdrop, this study investigates public opinions regarding anti-gay laws in Barbados, Guyana and T&T. The study aims to (1) determine the level of support for the current anti-gay laws in these countries and (2) evaluate the extent to which social and psychological factors (specifically socio-demographics, religion, interpersonal contact with homosexuals and beliefs about the origins of homosexuality) explain public support for these laws.

To date, the current literature suffers from a lack of research on support for gay rights in countries who still have laws penalising same-sex intimacy. Over the last decade, the literature on gay rights has focused on North America, and to a lesser extent European countries, who have abandoned their anti-gay legislations and are currently leading the fight for equality. By focusing on these three Caribbean states, this study adds to the sparse body of literature on attitudes towards gay rights in countries who currently have bans on same-sex intimate behaviours. Indeed, in the age where the fight for gay rights is universal, activists, academics and policymakers are not just concerned about gay rights in the West, but around the world. Hence, while this study is important for policymakers in the Caribbean, it should also be of interest to academics and practitioners outside these countries.

The rest of this document is organised as follows: the next section provides a brief background on anti-gay laws in Barbados, Guyana and T&T. This is followed by a description of the data and statistical method. The penultimate section presents the empirical results, and then finally, the study concludes.

## Background

### The Anti-gay Laws of Barbados, Guyana and T&T

The anti-gay laws in Barbados, Guyana and T&T can be generally classified as relics of British imperialism. Particularly, during the Middle Ages, male homosexuality in England was viewed as perverting the state and regarded as offences against God (Nichols 1984). Needless to say, Barbados, Guyana and T&T did not escape their colonial masters’ condemnation of homosexuality. Colonial legislators believed that inflicting such laws could bring European morality to these uncivilised colonies (Gupta and Long 2008). Britain exported its views on sexuality to its colonies (LaFont 2001) and so, these countries were subjected to the 1861 Offences to the Person Act–which carried a penalty of imprisonment for the ‘abominable crime of buggery’ (that, is, anal sex)–and later, the 1885 Criminal Law Amendment Act, which introduced penalties for acts of ‘gross indecency’ between men. It should be noted that while the 1885 laws gave no formal definition of what exactly constituted gross indecency, in practice, acts of gross indecency have often been interpreted as all intimate acts between men other than anal sex (Waites 1998).

While the anti-gay laws were largely imposed on Barbados, Guyana and T&T during colonialism, today, these laws are often seen as representative of their culture. Several decades after gaining their independence from England,^1^ these three countries still have laws policing sexuality. As shown in Table 1, penalties for engaging in private consensual anal sex are quite serious. It should be noted that the bans on anal sex are not specified to be limited to acts between males; hence, technically, anal sex between a man and woman is also a criminal offence. However, in these countries, individuals tend to use the terms ‘decriminalisation of buggery’ and ‘decriminalisation of homosexuality’ interchangeably (Abramschmitt 2008), hinting that the laws are widely perceived as condemnations of male homosexuality, rather than the act of anal sex itself (AIDS-Free World 2010; Gaskins 2013).

**Table 1:** Anti-gay laws in Barbados, Guyana and Trinidad and Tobago—maximum penalties for private consensual acts between adults

Country	Legislation	Maximum penalty
Provisions for anal sex		
Barbados	Section 9 of the 1992 Sexual Offences Act	Life imprisonment
Guyana	Section 354 of the 1998 Criminal Law Offences Act	Life imprisonment
T&T	Section 13 (1b) of the Sexual Offences (Amendment) Act, 2000	25 years imprisonment
Provisions for acts of gross/serious indecency		
Barbados	Section 12 (1) of the 1992 Sexual Offences Act	10 years imprisonment
Guyana	Section 352 of the 1998 Criminal Law Offences Act	2 years imprisonment
T&T	Section 16 (1b) of the Sexual Offences (Amendment) Act	5 years imprisonment
Homosexuals banned from entering the country		
T&T	Article 8 (1e) of the Immigration Act, 1995	N.A.

The laws on gross/serious indecency, however, are a bit more specific. In Guyana, the laws largely resemble the 1885 Criminal Law Amendment Act, and prohibits acts of gross indecency between men. Meanwhile, in T&T, acts of serious indecency are limited to same-sex acts, as Section 16(2) of T&T’s Sexual Offences (Amendment) Act, 2000 states that the penalties shown in Table 1 do not apply to acts of serious indecency committed in private between a husband and wife or a man and woman above the age of 16. Barbados is the only country not to explicitly target any same-sex intimate acts: unlike its Guyanese and T&T neighbours, acts of serious indecency are not specific to gender or sexual orientation. But, in spite of the gender neutrality of Barbados’ laws on acts of serious indecency, like the buggery laws, they are often mischaracterised as applying to individuals of a specific sexual orientation. Thus, the laws have symbolic power and lends to the marginalisation of homosexuals.

At first glance, T&T appears to be the most lenient in its legislation, as engaging in anal sex does not carry a (maximum) penalty of life imprisonment (Table 1). However, T&T is the only state to have increased its penalty for anal sex in the last three decades, moving from 5 years imprisonment prior to 1986 to 10 years under the Sexual Offences Act 1986 and finally, to 25 years in 2000. Also unique to the case of T&T is Article 8 of its Immigration Act that bans homosexuals from entering the country.

It should be noted that the anti-gay laws in these three countries are rarely ever used to police consenting private adult sexual activities. In fact, the laws are—to a large extent—unenforced. Thus, if same-sex conduct is rarely penalised, why keep the laws in place in the face of international pressures? It is possible that the laws are anchored by public support for the laws. In what follows, I evaluate the extent of public support for the laws and evaluate the factors correlated with support for said laws using a statistical model.

## Method

To undertake this study, I use secondary data attained from the 2013 Caribbean Development Research Services (CADRES) Attitudes Towards Homosexuals Survey, which was carried out in Barbados, Guyana and T&T. The Guyana and T&T surveys were funded by the UK Foreign and Commonwealth Office, while the Barbados survey was financed by the HIV/AIDS Commission in Barbados. The surveys were designed and administered by CADRES.

As noted in the CADRES reports (Caribbean Development Research Services 2013a, b, c), these national surveys employed stratified random sampling, with the primary strata being age and gender in each country. Interviewers were (randomly) assigned areas associated with the polling districts in each constituency, that is, the sub-divisions of the countries normally used for electoral processes (voting). The interviewers then randomly selected households in their assigned polling districts to interview. The survey was largely administered via face-to-face interviews, though interviewers complied with any requests from respondents to complete the survey themselves.

A total of 2871 adults (age 18 and over) were surveyed: 830 Barbadians, 1034 Guyanese and 1007 persons from T&T. This study focuses on the responses of those individuals who listed their sexual orientation as straight/heterosexual, and once missing observations were removed from across the dependent and independent variables, the estimation sample stood at 2165. Throughout the study, the reduced sample is used.

### Dependent Variables—Support for the anti-gay laws

Support for the laws, the key outcome variable, refers to whether or not respondents think the current laws should be maintained and/or enforced. Specifically, they are based on the following questionnaire items:

Presently, the laws of (insert country name here) outlaw the act of Buggery/Sodomy, whether between two men or a man and a woman and regardless of whether the act is in public or private, consensual or forced. Do you generally support the maintenance of this law?

- Yes, I think the Buggery/Sodomy laws should be maintained
- No, I think the Buggery/Sodomy laws should be changed (removed or modified)
- I am unsure/prefer not to say how I feel about this law

Currently, the laws of (insert country name here) with respect to Buggery/Sodomy are NOT being enforced (except in instances of forced sex/rape). Do you think that the state should enforce these laws by investigating and prosecuting persons who engage in these acts (by consent)?

- Yes, start enforcing the laws
- No, continue with non-enforcement
- Unsure/prefer not to say

**Fig. 1:**
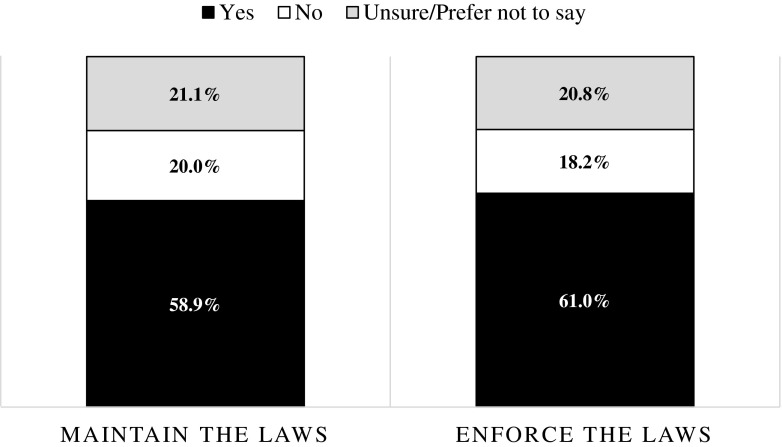
Support for the anti-gay laws. Sample size, 2165

**Fig. 2:**
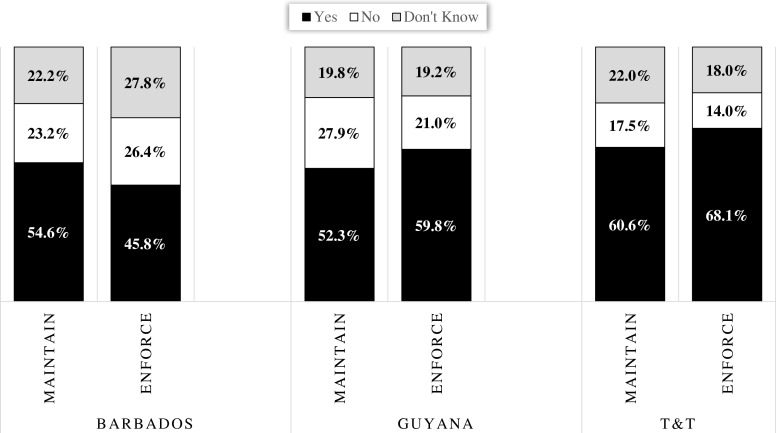
Public support for the laws by country. Sample size, 2165

The first aim of this paper was to determine the level of support for these laws. The distributions of the responses to the CADRES survey are shown in Fig. 1. A majority of respondents seem to support the current legislation—58.9 % think the laws should be maintained as is, and a slightly higher portion wants the laws enforced (61.0 %). Interestingly, a significant share of participants (roughly 21 %) said they were unsure or declined to state their opinions about the laws. Looking at public support by country (Fig. 2), a similar story emerges: in each country, a majority of persons support the laws. However, there is some evidence of country effects, with persons residing in T&T showing the highest support for both the maintenance and enforcement of the laws.

Further evaluation of the data (Table 2) revealed that some individuals, though stating the law should be changed or modified, opted for enforcement (7.4 %). It is possible that these persons may not agree with the punishments associated with the anti-gay laws or the fact that anal sex between opposite sex persons is also penalised, and so, think the laws should be changed but not abolished. Another 5.9 %, though opting for the laws to be maintained, did not want authorities to begin enforcing the laws. Arguably, this subset of individuals may prefer the law as a symbol—to persuade or prevent, not punish. Meanwhile, 10.2 % of the sample wanted the laws changed and also desired that non-enforcement be continued. This possibly represents persons who do not support the laws. Overall, there is a clear resistance to legislative changes, with a plurality of individuals (48.8 %) backing both the retention and enforcement of the laws.

**Table 2:** Public support for anti-gay laws—cross tabulations

			Enforce the laws	
Maintain the laws	Start enforcing the laws	Continue with non-enforcement	I am unsure/prefer not to say	Total
Maintain the laws	48.8 %	5.9 %	4.2 %	58.9 %
Laws should be changed or modified	7.4 %	10.2 %	2.4 %	20.1 %
I am unsure/prefer not to say	4.8 %	2.1 %	14.2 %	21.1 %
Total	61.0 %	18.2 %	20.8 %	

Sample size, 2165

### Independent Variables

Figures 1 and 2 suggest that in general, there is high support for the maintenance and enforcement of the anti-gay laws in these countries. The next logical question is, who is likely to support these laws? The empirical literature to date is very scant in terms of attitudes towards homosexuals and their respective rights in the Caribbean, which in turn may largely be due to a lack of national surveys on the issue. As such, this paper strongly relies on the previous literature (based on North America and Europe) and data available in the CADRES surveys to build an empirical model of public support for anti-gay laws in Barbados, Guyana and T&T. Specifically, public support for the laws is modelled as a function of religion, socio-demographics, beliefs about the origins of homosexuals and interpersonal contact with homosexuals. The following subsections describe the variables used and information on the distribution of the variables is given in Table 3. It is important to point out that CADRES measured all survey items categorically; hence, all variables are categorical in the study.

**Table 3:** Distribution of independent variables

Variables	Shares in percent
Religious identity	
Evangelical Christian (base)	40.1
Non-evangelical Christian	26.6
Muslim	4.8
Hindu	11.1
Other religion	10.7
Not religiously affiliated	2.9
No response	3.9
Religious participation	
Passive (base)	42.5
Active	48.5
Unsure/won’t say	9.0
Source of views on sexuality	
Non-religious sources (base)	40.5
Religion	48.7
Unsure/won’t say	10.9
Age	
18–30 (base)	35.8
31–50	34.1
51+	30.1
Gender	
Male (base)	49.3
Female	50.7
Education	
No tertiary education (base)	72.9
Tertiary education	27.1
Racial identity	
Black (base)	54.7
White	1.4
Indo Caribbean	19.7
Mixed	18.7
Other	4.0
No response	1.6
Marital status	
Single (base)	44.5
Married	34.4
Common-law marriage	6.7
Divorce/separated/widowed	12.4
Won’t say	2.0
Country of residence	
T&T (base)	40.6
Barbados	29.6
Guyana	29.9
Interpersonal contact	
No gay friends (base)	56.7
Gay friends	37.6
Prefer not to say	5.7
Attribution	
Choice (base)	34.9
Born gay	17.0
Other cause (poor religious values, psychological trauma, etc.)	34.6
Unsure/prefer not to say	13.5

Sample size, 2165

#### Religion

A principal justification for opposing LGBT rights is religious morality (Herek 1991). The sacred texts of most mainstream religions contain scriptural passages that are frequently interpreted as condemnations of homosexuality. And so, some religious individuals argue that they cannot support laws extending civil rights to homosexuals as this would violate their personal moral standards (Herek 1991). In fact, one of the most consistent findings in the academic literature is that religious individuals tend to be less accepting of homosexuals than the religiously unaffiliated, and by extension, less willing to grant them civil liberties (Walls 2010; Whitley 2009). Moreover, the impact of religious beliefs and affiliations tends to be greatest among those individuals for whom religion is more salient or who are more involved in their religion (Barringer et al. 2013; Becker and Scheufele 2009; Gerhards 2010; Hayes and Dowds 2015; Rowatt et al. 2009, 2009; Whitehead and Baker 2012).

Unfortunately, the CADRES data does not explicitly identify popular measures of religiosity used in the literature. But, three of the survey’s items can be used to test the role of religion in shaping anti-gay attitudes. First, respondents were asked about their religious identities, which in this study are presented as a 7-category variable: (1) evangelical christians (the reference category), (2) non-evangelical christians, (3) muslim, (4) hindu, (5) other religion, (6) religiously unaffiliated and (7) no response. Second, participants were asked to identify their religious participation and were given three choices: (1) ‘active’ (which I use as the reference category), (2) ‘passive’ and (3) ‘unsure/won’t Say’. Finally, respondents were asked about the source of their views on human sexuality. Respondents were asked to choose from (1) non-religious sources (such as popular culture or socialisation), which served as the reference category, (2) religion or (3) unsure/prefer not to say.

#### Socio-demographics

Some segments of the population tend to have higher levels of hostility towards homosexuals, and by extension, gay rights (Andersen and Fetner 2008b; Hayes 1997). For instance, it is widely accepted that university graduates tend to be more approving of homosexual relations than those who did not attend college (Loftus 2001; Ohlander et al. 2005). There is also evidence to suggest that age matters—younger persons tend to be more accepting of homosexuals—though it is unclear as to whether these differences result from birth cohort effects, period effects or both (Andersen and Fetner 2008a). The literature also suggests that gender matters. Specifically, men are more likely than women to manifest sexual prejudice (Hayes 1997; Kite and Whitley 1996; Whitley 2001) possibly due to the fact that males place heavier emphasis on traditional gender roles than women (Guittar and Pals 2014). There are a few studies linking being married—an event associated with traditional lifestyles—to more conservative attitudes (Brumbaugh et al. 2008; Herek and Capitanio 1995); while others have looked at racial variations (Herek and Capitanio 1995; Jenkins et al. 2009; Lewis 2003; Negy and Eisenman 2005). Following previous works, this study controls for the impact of education, age, gender, marital status and race. Also, as shown previously in Fig. 2, average support for the laws differed slightly across these three countries. Hence, I also control for potential differences in the level of support for the anti-gay laws across the three countries.

The education and gender variables are in binary form, where those who did not attend a tertiary institution serve as the reference category for the education variable and the base for the gender variable is males. Age is recorded categorically using three distinct age groups: 18–30, 31–50 and 51 and over, where persons aged 18–30 serve as the contrast. Race is divided into six categories: (1) black (base category), (2) white, (3) indo Caribbean, (4) mixed, (5) other and (6) no response, while marital status is a five category variable: (1) single (base), (2) married, (3) common-law marriage, (4) divorced/separated/widowed and (5) won’t say. Finally, cross-country differences are modelled via a three category variable, where T&T is arbitrarily chosen as the reference category.

#### Interpersonal Contact

In addition to demographics and religion, interaction with homosexuals has been shown to be a powerful predictor of attitudes towards homosexuals (Herek and Capitanio 1996; Herek and Glunt 1993; Lewis 2011). Specifically, the literature suggests that knowing someone who is lesbian or gay is positively related to acceptance of homosexuality—a finding in line with the contact hypothesis (Allport 1954), which states that intergroup contact helps reduce prejudice against an out-group. Here, the interpersonal contact variable is defined as whether or not individuals have homosexual friends. It is a three category variable: (1) the respondent has no gay friends (base outcome); (2) the respondent has gay friends and (3) the respondent choose the ‘don’t know/prefer not to say’ option in the survey.

#### Beliefs About the Origin of Homosexuality

Attribution style is also presumed to be a key determinant of public support for anti-gay legislation. Attribution theory, in its most basic form, supposes that the perceived cause or controllability of behaviour influences how individuals view a stigmatised group or behaviour. Indeed, the crux of the argument for gay rights concerns whether homosexuality is a choice or genetic (Whitehead 2010). Specifically, heterosexuals who believe that homosexuality is not innate are more likely to condemn homosexuals and oppose gay rights (Haider-Markel and Joslyn 2008; Lewis 2009; Whitehead 2010). In the CADRES survey, individuals were asked to select their belief about the causes of homosexuality. The variable in this study consists of four categories: (1) homosexuals choose to be that way (reference category); (2) some persons are just born that way; (3) other cause (poor religious values, psychological drama, bad parenting, etc.) and (4) unsure/prefer not to say.

### Statistical Model

For the empirical investigation, the dependent variables are dichotomised to form two variables defined as: 1) ‘Maintain’, which takes on a value of 1 if the respondent supports the maintenance of the laws and 0 otherwise (that is, thinks the laws should be abolished/changed or were unsure/preferred not to say); and 2) ‘Enforce’, which takes on a value of 1 if the respondent agrees that the laws should be enforced and 0 otherwise. In this way, the paper is specifically modelling heterosexual support for the anti-gay laws.

Since the coded dependent variables are binary in nature, one could estimate two separate probit or logit models for each of the dependent variables. However, the cross tabulations (Table 1) hints there is a high degree of interrelatedness between the dependent variables. In fact, the tetrachoric correlations (that is, the correlation coefficient for binary variables) was estimated at 0.75 with a corresponding *p* value that was less than 0.001, lending further evidence to the hypothesis that the dependent variables are strongly correlated. As such, a bivariate probit model is used to investigate heterosexual support for the laws, as these models allow for correlation between two binary dependent variables.

It should be noted that the ‘bivariate’ term in bivariate probit regression refers to the number of binary dependent variables, and not the number of independent variables. Typically, this model is used when two binary dependent variables are correlated and this correlation is believed to persist even after regressing the two dependent variables on a set of independent variables. Essentially, the bivaraite probit regression estimates the model within a system (i.e. estimate all regressions jointly), thus permitting cross-equation error correlations and leads to more efficient estimates than that obtained from regressing two separate models.

### Empirical Results

Table 4 presents the results. The bivariate probit model was estimated with all the independent variables in the model; hence, one is able to see the impact of each variable ceteris paribus. Bivariate probit models are characterised by their cross-correlation terms, that is, the correlation between the two dependent variables that remains even after regressing on the predictors. The cross-correlation term is 0.765, and the null hypothesis that the errors are not correlated (*H*_0_: *ρ*_12_ = 0), is strongly rejected, suggesting that a system approach to estimation is more appropriate than two individual probit models.

**Table 4:** Bivariate probit model estimates of heterosexual support anti-gay laws—average marginal effects reported

	Maintain	Enforce
Religious identity		
Evangelical Christian (reference)		
Non-evangelical Christian	0.001	−0.023
Muslim	0.019	−0.006
Hindu	−0.008	0.017
Other religion	−0.029	0.028
Not religiously affiliated	−0.065	−0.192^**^
No response	−0.146^**^	0.006
Religious participation		
Passive (reference)		
Active	0.043^+^	0.040^+^
Unsure/won’t say	−0.002	0.036
Source of views on sexuality		
Non-religious source (reference)		
Religion	0.065^**^	0.093^***^
Unsure/won’t say	−0.090^*^	−0.019
Age		
18–30 (reference)		
31–50	0.031	−0.025
51+	0.048	−0.003
Gender		
Male (reference)		
Female	−0.015	−0.004
Education		
No tertiary education (reference)		
Tertiary education	0.035	−0.010
Racial identity		
Black (reference)		
White	−0.245^**^	−0.262^**^
Indo Caribbean	−0.019	0.015
Mixed	−0.013	−0.026
Other	−0.029	−0.098^+^
No response	−0.319^***^	−0.035
Marital status		
Single (reference)		
Married	0.011	0.029
Common-law marriage	0.071^+^	0.062
Divorce/separated/widowed	−0.020	0.007
Won’t say	−0.067	−0.062
Country of residence		
T&T (reference)		
Barbados	−0.005	−0.188^***^
Guyana	−0.079^**^	−0.085^***^
Interpersonal contact		
No gay friends (reference)		
Gay friends	−0.075^**^	−0.041^+^
Prefer not to say	−0.188^***^	−0.023
Attribution		
Choice (reference)		
Born gay	−0.140^***^	−0.129^***^
Other cause of homosexuality	0.001	0.011
Unsure/prefer not to say	−0.080^**^	−0.136^***^
ρ_12_	0.765^***^	

^***^Indicates statistical significance at the 0.1 % level; ^**^Indicates statistical significance at the 1 % level; ^*^Indicates statistical significance at the 5 % level; ^+^Indicates statistical significance at the 10 % level

A key concern among researchers is the substantive and practical significance of the coefficients provided by the bivariate probit model: while the coefficients provided are a good indication of the sign and statistical significance of the predictors, their interpretation is not intuitively appealing. As such, I opted to calculate the average marginal effects, which are easier to interpret (Cameron and Trivedi 2010; Greene 2012). The average marginal effects (AMEs) are quite similar to the coefficients estimated in simple regression models. In this paper, the AMEs indicates the average percentage point differences in probability between the reference category of a variable and the other categories of that variable.

Looking first at the case of religion, in the ‘Maintain’ equation, there is no evidence to suggest that religious identity matters. However, in the ‘Enforce’ equation, there appears to be a striking difference between the secular and the sacred. In this sample, the probability that the religiously unaffiliated will support law enforcement is 20.9 percentage points lower than those individuals who identified as Evangelicals. Since there is no evidence of significant differences between religious denominations, the results suggest that the religiously unaffiliated are less likely to support enforcement than those who identify with a religion. With respect to religiousness, there was some (albeit weak) evidence that the more involved an individual is in their religion, the more likely they are to support the laws. The probability that an individual who is actively involved in their religion will want the laws maintained is roughly 4.5 percentage points greater than that of persons passively involved in their religion. Similar differences are found for the law enforcement category. However, in both equations, the religious participation variable was only significant at the 10 % level of testing. Meanwhile, the probability that an individual whose views on sexuality have a theological base will support the statutes is greater than that of an individual whose views on sexuality were not religiously inspired. In terms of law retention, the aforementioned difference in probabilities is 8.0 percentage points; for enforcement, the difference is 10.6 percentage points.

Turning now to the socio-demographics, there is very little evidence of heterogeneity across the socio-demographics. For instance, gender, age and education are statistically insignificant across the board. However, there is some evidence that race, marital status and country of residence matters. Specifically, white Caribbeans appear to be least supportive of the laws while persons in a common-law marriage appear most likely to want the laws maintained. There is also some evidence that place of residence matters, as Guyanese respondents seem least likely to state that the laws should be maintained, while respondents from T&T appear to offer the most support for enforcement.

The results also suggest that beliefs about the origins of homosexuality is a strong predictor of public support for gay rights. In line with previous research, the results suggest that individuals who believe that homosexuality is innate are less supportive of the laws than those who believe otherwise. Support for the anti-gay laws also seems susceptible to intergroup contact, as respondents with affective ties with homosexuals appear less supportive of the laws than those without.

The results presented thus far have identified the factors influencing the marginal probability that a respondent wants the laws maintained, and the marginal probability that someone wants the laws enforced. However, as shown in Table 1, a large portion of the sample supported both the maintenance and enforcement of the laws. There were also some persons who initially said the laws should be maintained, but when asked about enforcement (a stricter category) either said no, or stated that they were unsure. This raises the question, who is likely to want both the maintenance and enforcement of the laws? And, who is likely to opt for maintenance, but then either be against enforcement or not sure about it? One of the key benefits of the bivariate probit model is that one is able move beyond the marginal probabilities model and evaluate other types of probabilities via the estimated system. In this study, I first evaluate the factors influencing the probability that someone wants the laws enforced and maintained, that is, the joint probability that Maintain = 1 and Enforce = 1. I then seek to model the following conditional probability: Pr (Enforce = 0 | Maintain = 1) that is, the probability that the respondent will not support the enforcement of the anti-gay laws, given that the respondent supported law retention. Since analysis of the joint/conditional probabilities are done within the system, it is much more efficient than arbitrarily creating a variable from the survey data. The results for the joint and conditional probabilities are given in Table 5.

**Table 5:** Joint and conditional models—average marginal effects reported

	Joint probability: Pr (enforce = yes, maintain = yes)	Conditional probability Pr (enforce = no or don’t know|maintain = yes)
Religious identity		
Evangelical Christian (reference)		
Non-evangelical Christian	−0.011	0.021
Muslim	0.007	0.014
Hindu	0.004	−0.018
Other religion	−0.004	−0.036
Not religiously affiliated	−0.140^*^	0.164^*^
No response	−0.093^+^	−0.065^*^
Religious participation		
Passive (reference)		
Active	−0.045^*^	0.017
Unsure/won’t say	−0.029	−0.016
Source of views on sexuality		
Non-religious source (reference)		
Religion	0.085^***^	−0.052^**^
Unsure/won’t say	−0.059^+^	−0.027
Age		
18–30 (reference)		
31–50	0.005	0.036^+^
51+	0.029	0.018
Gender		
Male (reference)		
Female	−0.011	−0.003
Education		
No tertiary education (reference)		
Tertiary education	0.014	0.025
Racial identity		
Black (reference)		
White	−0.251^**^	0.133
Indo Caribbean	−0.003	−0.021
Mixed	−0.021	0.017
Other	−0.069	0.080^+^
No response	−0.236^**^	−0.102^**^
Marital status		
Single (reference)		
Married	0.021	−0.020
Common-law marriage	0.073^+^	−0.023
Divorce/separated/widowed	−0.007	−0.015
Won’t say	−0.067	0.025
Country of residence		
T&T (reference)		
Barbados	−0.107^***^	0.170^***^
Guyana	−0.087^***^	0.033^+^
Interpersonal contact		
No gay friends (reference)		
Gay friends	−0.063^**^	0.003
Prefer not to say	−0.129^**^	−0.060^*^
Attribution		
Choice (reference)		
Born gay	−0.142^***^	0.049^*^
Other cause of homosexuality	0.011	−0.005
Unsure/prefer not to say	−0.115^***^	0.087^**^

^***^Indicates statistical significance at the 0.1 % level; ^**^Indicates statistical significance at the 1 % level; ^*^Indicates statistical significance at the 5 % level; ^+^Indicates statistical significance at the 10 % level

With respect to the joint probability model, the results suggest that the individual most likely to support both the retention and enforcement of the anti-gay laws: (1) is religious, that is, religiously affiliated, active in his/her religion and his/her main views of sexuality have a theological base; (2) does not identify as a white Caribbean; (3) is in a common-law marriage; (4) resides in T&T; (5) has no gay friends and (6) believes homosexuality is a choice. Turning now to the conditional probability, the persons most likely to showcase symbolic law support: (1) is religiously unaffiliated and did not cite religion as their main source of views on human sexuality; (2) in the 31–50 age group; (3) chose the ‘other’ category when asked about their racial identity; (4) resides in either Barbados or Guyana and (5) believes persons were born gay or unsure about the origins of homosexuality.

## Discussion

This study sought to evaluate heterosexual support for unenforced bans on homosexual behaviours in three Caribbean states: Barbados, Guyana and T&T. A majority of the sample supported the anti-gay laws, and the greatest level of support came from respondents in T&T. This implies that there could be some public backlash should legislators in these states bow to international pressures and remove the laws. In fact, with a majority of persons supporting law retention in its current form, lawmakers could be hesitant to change such laws and stand outside of public favour. However, should policymakers decide to bow to international pressures, an understanding of the factors driving said support would be imperative to limit backlash.

In the second stage of the study, I evaluated the factors correlated with support for the anti-gay laws. First, the results suggested that religion plays a role in sexual prejudice. Specifically, there is evidence that individuals who were actively involved in a religion and whose views on human sexuality were shaped by religion were more likely to support the laws than persons passively involved in their religion or those with non-religious views on human sexuality. Also, the religiously unaffiliated were less likely to support both maintenance and enforcement of the laws, but more likely to want the laws in place as a symbol—that is, maintained but not enforced. This finding is somewhat expected. In their guidelines for moral living, many religious scripts stress the importance of sex for procreation. Homosexual relations do not fit into this paradigm and as such it is deemed as ‘unnatural’, ‘immoral’ and even ‘an abomination’ (Hough 2004; Wilson 1971). Thus, it is not surprising that public support for bans on same-sex intimacy is greatest among those individuals for whom religion is more salient.

Interestingly, while the religious tend to be more disapproving of the laws, there is no evidence of differing levels of support across religious denominations. This is a contrast to previous studies that reported significant differences in the extent to which persons of different religions condemn homosexual behaviours (Adamczyk and Pitt 2009; Cadge et al. 2008; Hooghe et al. 2010; Olson et al. 2006; Schulte and Battle 2004; Sherkat et al. 2011). Taken at face value, it would seem as though in these states, there is some sort of religious syncretism in terms of attitudes towards the anti-gay laws.

The findings also suggest that demographics play a limited role in explaining support for anti-gay laws in the Caribbean, with race and country of residence being the most consistent demographics. Somewhat surprising was the fact that education did not play a significant role in predicting any of the measures of heterosexual support of the laws, and that age only mattered in the conditional probability model. This marks some deviations from the current literature, as several studies based on the US and Europe often cite substantial differences in hostility towards homosexuals by age and education. At first glance, the insignificance of education and weak evidence of an age impact may seem surprising. However, one must consider the hypothesised reasons why age and education matter for sexual prejudice. With respect to education, early work (Quinley and Glock 1979; Vogt 1994) suggested that higher education reduces prejudice by providing more information about minorities, teaching persons to recognise prejudice and understand its negative impacts, and by providing the cognitive skills to reject prejudice. These efforts tend to be exemplified in Western colleges and universities, who in recent decades have employed intentional interventions to reduce sexual prejudice. For instance, there are several rules and regulations in place that explicitly prohibit discrimination on the basis of sexuality, as well as curriculums that teach about sexual diversity in North American and European schools and universities. Against a backdrop of state-sponsored homonegativity, it would seem plausible to assume that the intensity of efforts taken to eliminate sexual prejudice in Western educational institutions are not present in tertiary institutions in Barbados, Guyana and T&T. Thus, it is possible that attending a local university may not lead to a significant change in an individual’s attitude towards the anti-gay laws. With respect to age, changing socialisation patterns has been cited as a key reason for the observed generational gaps in Western attitudes towards homosexuality as in recent decades, homosexuals have increasingly been presented in a positive light, particularly in the media (Baunach 2011; Lewis and Gossett 2008; Sherkat et al. 2011). However, in these three states, homosexuals are still seen in a very negative light. It is then plausible that the disapproval of homosexual acts has been passed on to succeeding generations.

The results also imply that having a gay friend has a sizeable impact on law support. Generally, persons with a gay friend are less likely to support the maintenance and/or enforcement of the laws. Taken at face value, it would seem as though socialising with a person who is a lesbian or gay male may help to break negative stereotypes about gay persons, and increase support for gay rights. However, in these three states where traditional cultural norms of sexuality are prevalent and there are no laws preventing discrimination on the basis of sexuality, homosexuals may opt to remain in the closet for fear of condemnation. This then limits the extent to which interpersonal contact could become a key channel through which prejudice is negated. It is also important to note that to some extent, the observed correlation between interpersonal contact and law support may be due to selection effects. Gay men and lesbians are more likely to reveal their sexual orientation to heterosexuals from whom they expect a positive response (Herek and Glunt 1993). Hence, the observed correlation may be due to the fact that persons have homosexual friends because they had pre-existing favourable attitudes towards homosexuals (Hans et al. 2012).

Finally, with respect to the beliefs about the origin of homosexuality, the empirical model suggests that heterosexuals who believe that persons are born gay were less likely to support the enforcement and/or maintenance of the laws. They were also more likely to only want the laws maintained, but not enforced. This is not surprising. One of the inherent assumptions in criminal law is that persons are responsible for their actions. Thus, persons who believe that homosexuality is innate would logically be less likely to believe that same-sex intimate acts should be penalised. At face value, it would appear that changing mind-sets about the controllability of homosexual behaviours could aid in reducing support for the laws. However, this is by no means an easy task. Though there has been an increase in studies linking genetics to homosexual behaviour in recent years, there is currently no overall consensus in the scientific community regarding the biological basis of homosexuality (Haider-Markel and Joslyn 2008). The lack of a consensus means that persons could be less likely to change their views about the unnaturalness of homosexuality, and so, continue to reject extending rights to homosexuals. However, attributions could be changed in the future, particularly in the face of a unified scientific view on the genetics of homosexuality.

While this study sheds some quantitative light on the drivers of heterosexual support for anti-gay laws, it is not without its limitations. First, there are a host of variables that the literature has identified as key determinants of attitudes towards homosexual rights (such as attitudes about sex roles, conservatism and levels of authoritarianism) whose impact could not be captured in this study. Another limitation concerns the measures of religiousness used. Unfortunately, the religious participation variable was largely subjective; surely, interpretations of ‘active’ and ‘passive’ would vary across respondents. The research could have benefited from more standardised measures of religiosity such as frequency of worship, attendance or the importance of religion to one’s life. Finally, the study only included three Commonwealth Caribbean countries, and this was largely due to data availability. Currently, there are eight other Commonwealth Caribbean states (that is, Antigua and Barbuda, Belize, Dominica, Grenada, Jamaica, St Kitts and Nevis, St Lucia and St Vincent and the Grenadines), who also inherited anti-gay laws from Britain and have opted to continue policing sexuality. An area of future research could be conducting research for a larger subset of Caribbean countries, should such data become publicly available.

## Footnotes

1 T&T gained independence from England in 1962 while Barbados and Guyana gained independence in 1966.
